# Multidisciplinary Management of a Pediatric Patient With Congenital Anomalies Syndrome Without an Identified Genetic Mutation

**DOI:** 10.7759/cureus.64530

**Published:** 2024-07-14

**Authors:** Sungmin Song, Obioha Louis Okoro

**Affiliations:** 1 Medicine, Trinity School of Medicine, Warner Robins, USA; 2 Pediatrics, Central Georgia Pediatrics, Georgia, USA; 3 Pediatrics, Piedmont Macon Medical Center, Georgia, USA

**Keywords:** multiple congenital anomalies, tracheostomy placement, gastrostomy tube, non-genetic, multidisciplinary management

## Abstract

Congenital anomalies syndrome without an identified genetic mutation often presents significant challenges in pediatric care, requiring coordinated efforts across multiple specialties. This case reports a 10-year-old female patient with complex medical conditions, which exemplifies the intricate nature of managing children, necessitating long-term follow-up and comprehensive care. This case report aims to provide an in-depth analysis of her medical journey, including various interventions like tracheostomy and G-tube placement, and management strategies employed to address her congenital anomalies and associated health issues.

## Introduction

The prevalence of multiple congenital anomalies (MCAs) varies but is reported to be around 15.1 to 26.3 per 10,000 births. This condition involves multiple birth defects affecting various parts of the body and can occur due to non-genetic or unidentified genetic factors [1]. Conditions like micrognathia, microtia, and congenital vertebral anomalies, although rare individually, often occur together as part of a broader syndromic presentation. The exact prevalence of such complex, multi-system congenital anomalies is not well-documented due to their rarity and variability.

A 10-year-old female with a history of multiple congenital anomalies presents a complex medical case involving respiratory, gastrointestinal, cardiovascular, and craniofacial issues. Born with micrognathia, microtia, and vertebral anomalies, she required a tracheostomy [2] for airway management and a gastrostomy tube [3] for feeding at a young age, which was successfully removed. This report discusses her medical history, diagnostic findings, and the multidisciplinary approach [4] to her treatment and ongoing management, highlighting the complexities and challenges in managing such cases.

## Case presentation

A 10-year-old female was born on December 28, 2013. She spent the first 10 days of her life in the neonatal intensive care unit (NICU) due to multiple congenital anomalies. Recognizing the need for a multidisciplinary approach, her doctors transferred her to Children's Healthcare of Atlanta in Georgia on January 7, 2014 (Table 1).

**Table 1:** Patient background

Status	Details
Immunization	Up-to-date
Family History	Lives with mother, father, grandmother, and one sister. Family history is positive for cancer, heart problems, and high blood pressure, but the patient does not have medical conditions such as microtia or micrognathia
Social History	Born full-term with an extended neonatal intensive care unit stay
Medical History	No history of blood transfusions or personal or family history of bleeding disorders

At Children's Healthcare of Atlanta, she underwent various examinations, including cardiac, pulmonary, and genetic tests [5]. A genetic test and microarray analysis were performed to identify chromosomal abnormalities, copy number variants, and genetic syndromes. Additionally, a TCOF1 gene test was conducted due to the patient's symptoms resembling those of Treacher-Collins syndrome [6]. However, the microarray [7] and DNA sequencing of the TCOF1 gene revealed no mutation that could explain her condition, and as a result, there is no unifying diagnosis for her at this time. She also received a bone-conduction Auditory Brainstem Response (ABR) test, which is commonly done for children with craniofacial anomalies in this patient population to assess auditory function. It showed normal bone conduction with bilateral microtia [8]. She received a gastrostomy tube (G-tube) placement for feeding on April 10, 2014, and a tracheostomy on January 9, 2014, to manage severe upper airway obstruction and chronic lung disease.

She was stabilized with proper management, and on April 22, 2014, she was discharged with her parents having received appropriate education for home management. The physical exam at the time of discharge showed that her average blood pressure over 24 hours was 89/46 mmHg, her temperature was 36.9°C, and her heart rate was 165 beats per minute. Her respiratory rate was 42 breaths per minute, and her saturation of peripheral oxygen (SpO2) was at 99%. She weighed 4.91 kg and her height was 59 cm. She was alert and active with stable vital signs.

A physical exam also showed clear breath sounds with no wheezing or crackles. Her heart sounds were normal, although a small atrial septal defect (ASD) was noted. Her head and neck examination revealed micrognathia and bilateral microtia with the absence of the right external ear. Gastrointestinally, she had a G-tube in place for feeding. Neurologically, she exhibited normal muscle tone, although her developmental milestones were slightly delayed for her age.

Following her discharge, she started outpatient care, including primary care, ENT, pulmonology, and gastroenterology. Her first visit to Dr. Okoro's primary care clinic was on April 28, 2014, six days after discharge from the NICU. At that time, she presented in the pediatric office with micrognathia, microtia, eczema, and neonatal primary apnea.

She continued to receive multidisciplinary care to manage her symptoms, including regular visits to pulmonologists, ENT specialists, gastroenterologists, and speech pathologists. She received various treatments, especially for her tracheostomy tube, which used a 3.0 Bivona [9]. Through this comprehensive care, she progressed to the point where she could eat orally, allowing the removal of her G-tube. Additionally, her atrial septal defect (ASD) closed.

Throughout her growth, she routinely visited her pediatrician and was closely followed by specialists in gastroenterology, pulmonology, and ENT. Despite some delayed milestones, she has been thriving well with proper management, including routine check-ups with vaccinations. In 2021, she no longer needed the tracheostomy tube to support her breathing, and it was removed on November 15, 2021 [10].

In 2024, she continued her regular visits to her pediatrician and maintained close follow-ups with pulmonology and ENT specialists. She continues to exhibit some developmental delays, including short stature and a BMI in the 5th percentile; however, she has been making significant progress. She visited The ENT Center of Central Georgia and Atrium Health for a follow-up on her tracheostomy removal and to discuss the possibility of closing the tracheostomy hole. During the visit, she was wearing a soft band BAHA (Bone Anchored Hearing Aid) to cover the tracheocutaneous fistula without any difficulties. Her physical exam was normal, revealing clear middle ears, and no evidence of cholesteatoma, postnasal drip (PND), cobblestoning, or lymphadenopathy. She will have a consultation with surgery to close the tracheostomy hole (Tables 2-3) [11].

**Table 2:** Most recent ENT findings (5/22/2024) in The ENT Center of Central Georgia

Exam	Findings	Details
Nose/Sinus	Normal	Mucosa: Bilateral: Normal. Septum: Normal. Inferior turbinate: Bilateral: Normal. External nose: Normal. Nares: Right: Normal, Left: Normal
Head/Face	Normal	Facial features: Normal
Ears	Comments	Stable bilateral microtia
Ears	Normal	Inspection - Right: Normal, Left: Normal. Pinna - Right: Normal, Left: Normal. Canal - Right: Normal, Left: Normal. TM - Right: Normal, Left: Normal
ENT Oral	Normal	Upper lip: Normal. Lower lip: Normal. Vestibule of mouth: Normal. Floor of mouth: Normal. Tongue: Normal. Buccal mucosa: Normal. Salivary glands: Normal. Hard palate: Normal. Soft palate: Normal. Uvula: Normal. Tonsils/Tonsillar fossae: Normal. Retromolar trigone: Right: Normal, Left: Normal. Posterior pharyngeal wall: Right: Normal, Left: Normal. Voice quality: Normal. Gag reflex: Present
Neck Exam	Comments	Stable tracheocutaneous fistula
Neck Exam	Normal	Inspection - Normal. Palpation - Normal. Parotid gland - Normal. Thyroid gland - Normal. Range of motion - Normal. Submandibular lymph nodes - Normal. Cervical lymph nodes - Normal

**Table 3:** Laboratory findings of the lipid panel test on 5/22/2024 in Atrium Health

Test	Value	Ref Range & Units
Cholesterol, Total, Lipid Panel	136	100 - 170 mg/dL
Triglycerides, Lipid Panel	51	20 - 129 mg/dL
HDL Cholesterol, Lipid Panel	59	45 - 60 mg/dL
LDL Cholesterol, Calculated	64	50 - 90 mg/dL
Non-HDL Cholesterol	77	30 - 144 mg/dL

## Discussion

Pathophysiology

**Figure 1:**
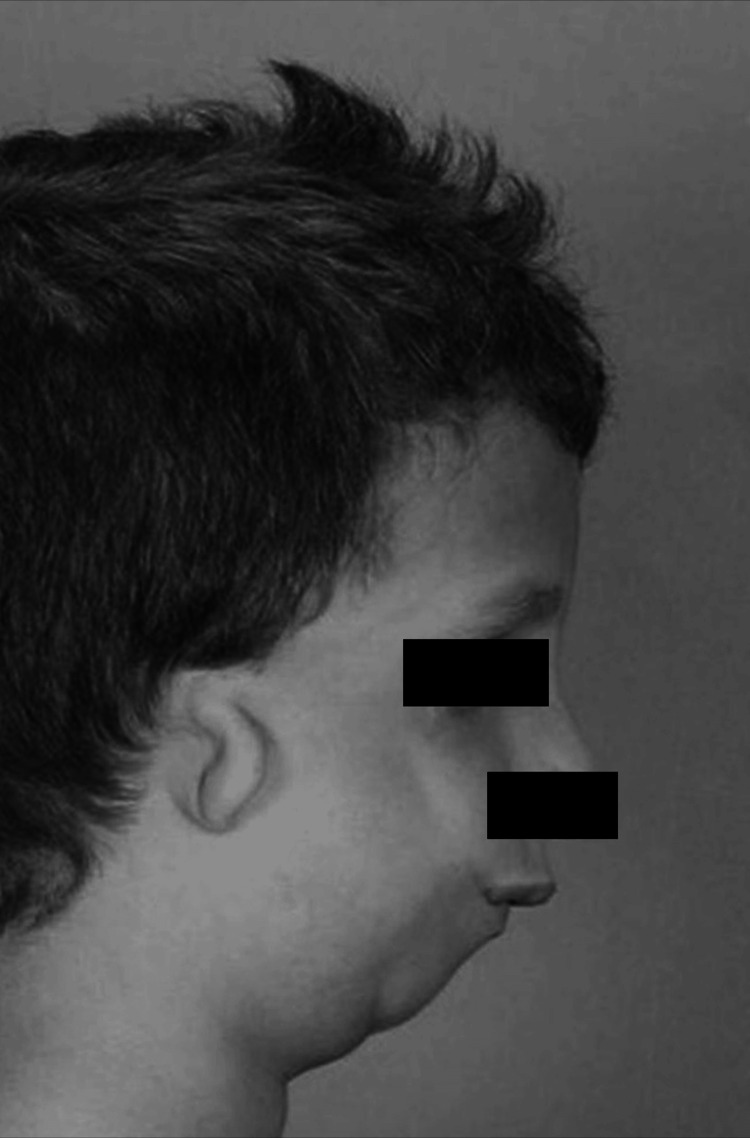
Illustration of microtia and micrognathia in a pediatric patient This figure does not belong to the patient in the present article. This is an example image that has been adapted from Ref. [12], which is an open-source article distributed under the terms and conditions of the CCBY 4.0 license.

Micrognathia, characterized by an undersized jaw, often presents challenges like airway obstruction, feeding difficulties, and dental issues. Similarly, microtia, an ear malformation often associated with the absence of an ear canal, can lead to conductive hearing loss, varying from unilateral to bilateral and in severity (Figure 1) [12]. Additionally, congenital vertebral anomalies may cause spinal instability, scoliosis, and other structural issues, impacting mobility and development. While these anomalies may arise from genetic conditions or occur in isolation, our case underwent genetic testing, including microarray analysis and a TCOF1 gene test, to identify chromosomal abnormalities, copy number variants, and genetic syndromes. However, the results returned negative, suggesting other potential contributors.

Multifactorial inheritance is one such aspect where a blend of genetic susceptibility and external influences, such as prenatal exposure to teratogenic substances or maternal infections during crucial developmental stages, could induce anomaly development, even in the absence of specific genetic mutations. Additionally, there's a possibility of unknown genetic variants that were not identified by the conducted genetic tests. These factors collectively emphasize the complexity of congenital anomalies and the need for comprehensive evaluation beyond genetic testing alone. Idiopathic cases, which account for approximately 50% of congenital anomalies, highlight the significant portion of cases where the underlying cause remains unknown despite extensive investigation [13].

Comparison with previous studies

Previous patients with multiple congenital anomalies, including craniofacial diseases, such as microtia and micrognathia, often have genetic mutations, though this is not always the case. The most well-known cause of craniofacial anomalies like these is Treacher-Collins syndrome, which presents with micrognathia and microtia due to mutations in the TCOF1 gene. This is why the NICU conducted genetic testing to confirm the diagnosis, which came back negative in this case [14]. Another similar case is the Pierre Robin Sequence, which is also a common cause of craniofacial anomalies, such as micrognathia, glossoptosis, and airway obstruction, due to genetically mutated 22q11.2 deletion [15]. Those two studies showed similar symptoms to this case but are based on genetic mutations. As mentioned, there are also non-genetic cases with micrognathia and microtia along with upper airway obstruction. These cases also involve negative microarray results and present with craniofacial anomalies. However, there are differences between this patient and my patient such as the presence of an atrial septal defect (ASD). This patient also requires multidisciplinary management and ongoing monitoring, similar to my patient [16]. With this comparison, we can highlight the importance of comprehensive genetic and clinical evaluation to diagnose and manage patients effectively.

Prognosis

The prognosis of the patient in the current case report is optimistic. The successful removal of her tracheostomy indicates significant respiratory improvement. The surgical option for closure of the tracheostomy hole is considered for the future. However, she will require ongoing monitoring and intervention for her other congenital anomalies. According to her pediatrician, she is thriving very well despite her delayed growth. With comprehensive multidisciplinary care, her quality of life can be optimized, though she will likely face ongoing challenges related to her congenital conditions.

## Conclusions

This case emphasizes the complexity of managing multiple congenital anomalies in pediatric patients. A multidisciplinary approach, involving regular follow-ups and coordinated care across various specialties, is essential for improving health outcomes and quality of life. Her case highlights the importance of early intervention, continuous monitoring, and adaptive management strategies in addressing the unique challenges presented by congenital anomaly syndrome. Due to advancements in medical services, her successful management was possible, highlighting the invaluable contributions of modern healthcare in addressing such intricate medical challenges.
